# Editorial: From Glycerol to Value-Added Products

**DOI:** 10.3389/fchem.2020.00069

**Published:** 2020-02-11

**Authors:** Mohamed Kheireddine Aroua, Patrick Cognet

**Affiliations:** ^1^Centre for Carbon Dioxide Capture and Utilisation, School of Science and Technology, Sunway University, Subang Jaya, Malaysia; ^2^Department of Engineering, Faculty of Science and Technology, Lancaster University, Bailrigg, United Kingdom; ^3^Laboratoire de Génie Chimique, Université de Toulouse, CNRS, INPT, UPS, Toulouse, France

**Keywords:** glycerol, green chemistry, catalysis, process, activation, added value bio-based products, electrochemical conversions

Increases in biodiesel production and the demand for oleochemical-based products have led to the generation of huge amounts of crude glycerol, which has given birth to new challenges regarding its sustainable use. Although there is a wide range of potential uses for crude glycerol, there are limited by its degree of purity, which affects its physical, chemical, and biological properties. The chemical transformation of glycerol has thus become a major point of interest for crude glycerol valorization. High added value products can be obtained from glycerol through different pathways, such as oxidation, carbonylation, reforming, acetalyzation, etherification, esterification, dehydration, hydrogenolysis, etc. Starting from a poly-hydroxylated molecule, all these chemical routes generally lead to complex mixtures and are not selective. In order to develop further industrial processes, progresses must be achieved to increase yield and selectivity, reduce reaction times, and ensure that work in media is as clean as possible. The catalyst choice is also of great importance since it impacts the selectivity. Heterogeneous ones must be preferred for an industrial process because they can be easily separated. One other important aspect is the quality of the starting glycerol; it has a great influence on the synthesis performance. This special issue gathers some contributions focused on recent advances in some key aspects of glycerol transformation processes: the crude glycerol purification prior to use for chemical transformation, the use of new synthesis media, the use of non-thermal activation techniques such as electrochemistry and plasma, as well as the synthesis, use, and characterization of new heterogeneous catalysts. These principles are applied to the optimization of the synthesis of key added-value products, such as glycerol carbonate, glycerol oleates, glyceraldehyde, acrolein, glycolic acid, and lactic acid. Process aspects are also considered, such as the purification process or fluidized bed technology.

This special issue is a collection of 3 critical reviews and 10 original research articles.

The use of an electron as a clean reagent is of great interest to the goal of transforming glycerol in added-value products in a sustainable manner. As direct electron transfer for a polyhydroxylated molecule like glycerol would lead to the creation of a variety of products, an indirect electrocatalytic process is envisaged in this special issue. Lee et al. have investigated the use of mixed carbon black-activated carbon electrodes for glycolic and lactic acid production. Glycolic acid was then obtained with good yield and selectivity. On the other hand, Coutanceau et al. have proposed an overview of different catalytic systems and conditions to control the products selectivity obtained from glycerol electrooxidation.

Catalyst deactivation is also a crucial issue to overcome on the road to a sustainable industrial process. Liu et al. have demonstrated the benefits of non-thermal plasma technology to avoid silice-supported silicotungstic acid catalyst deactivation during glycerol dehydration for acrolein production.

Glycerol purity is crucial for its further selective transformation into various products. Therefore, in order to valorize crude glycerol for synthetic purposes, a purification step is necessary. Abdul Raman et al. propose, in this special issue, a dual-step purification method that includes acidification and ion exchange operations, which allows it to reach a 98.2% purity.

This special issue also focuses on targeted molecules derived from glycerol. de Caro et al. have presented the recent advances concerning glycerol carbonate (GC) synthesis. Amongst the different routes, DMC and glycerol are good precursors, leading to GC through transcarbonation under mild conditions. Bruniaux et al. have reported the selective conversion of glycerol into 3-methoxypropan-1,2diol in mild yields. Al-Saadi et al. have investigated a reactive coupling that associates the transesterification of rapeseed oil into a fatty acid methyl ester and glycerol carbonate in a one-step process by introducing triazabicyclodecene guanidine as a catalyst.

Viable industrial chemical processes imply the use of heterogeneous catalysts for easy product recovery and catalyst recycling. Moreover, catalyst activity strongly relies on its physical properties, such as hydrophilicity/hydrophobicity, acidity, stability to water, etc. Muraza et al. have investigated the performances of natural zeolites and natural clays as low-cost catalysts. For a specific application, such as esterification with oleic acid, new catalysts must be developed, and these should exhibit good proprieties in terms of acidity and hydrophobicity. Kong et al. have developed, characterized, and studied hydrophobic zirconia-silica acid catalysts. They obtained 80% glycerol conversion together with 60% mono-oleate selectivity. Pt-based solid catalysts deposited on various supports have been studied by El Roz et al. when applied to the synthesis of glyceraldehyde from glycerol. The best activity was obtained for Pt/g-Al_2_O_3_, whereas best selectivity was obtained using Pt/SiO_2_. Sulphonic acid-functionalized copolymer beads were also synthetized, characterized, and used for solketal synthesis from glycerol. Al-Saadi et al. succeeded in optimizing the process using a two-step acetone feeding process. A technico-economic analysis revealed that this process could compete with the current industrial one. This reaction—the acetalization of glycerol through acid catalysis—was also investigated by Talebian-Kiakalaieh et al. They give, in this special issue, a comprehensive study of the impact of the different operating parameters—a prerequisite for biorefinery development.

To succeed in these industrial implementations, a chemical engineering approach has to be coupled with a chemical one. For this purpose, Katryniok et al. developed a two-zone fluidized bed reactor to carry out the gas-phase dehydration of glycerol to acrolein, using phosphotungstic acid supported on silica as a catalyst. The fluidization quality, the catalyst mechanical stability, and the influence of the operating conditions were successively studied, thus showing, for example, the crucial part the O_2_/glycerol ratio plays for the purposes of conversion and selectivity.

The contributions made to this special issue of "From Glycerol to Value-Added Products” underline the variety of the research work carried out in the field of the valorization of glycerol and processing of raw material at low cost, and they tackle both the reactional aspects as catalysis, processes, and even economic aspects. Many chemical applications have been covered in this special issue, thus showcasing all the potential of glycerol. We hope that the issue will inspire the readers to further contribute to this exciting field of glycerol valorization.

Finally, we would like to thank all authors for their valuable contributions to this special issue, and we wish them success in their research.

## Author Contributions

MA and PC thank all contributors for their original articles submissions to this special issue.

### Conflict of Interest

The authors declare that the research was conducted in the absence of any commercial or financial relationships that could be construed as a potential conflict of interest.

